# Lon Protease Is Important for Growth under Stressful Conditions and Pathogenicity of the Phytopathogen, Bacterium *Dickeya solani*

**DOI:** 10.3390/ijms21103687

**Published:** 2020-05-23

**Authors:** Donata Figaj, Paulina Czaplewska, Tomasz Przepióra, Patrycja Ambroziak, Marta Potrykus, Joanna Skorko-Glonek

**Affiliations:** 1 Department of General and Medical Biochemistry, Faculty of Biology, University of Gdańsk, Wita Stwosza 59, 80-308 Gdańsk, Poland; tomasz.przepiora@phdstud.ug.edu.pl (T.P.); patrycja.ambroziak@phdstud.ug.edu.pl (P.A.); 2 Intercollegiate Faculty of Biotechnology, University of Gdańsk and Medical University of Gdańsk, Abrahama 58, 80-307 Gdańsk, Poland; paulina.czaplewska@ug.edu.pl; 3 Department of Environmental Toxicology, Faculty of Health Sciences with Institute of Maritime and Tropical Medicine, Medical University of Gdańsk, Dębowa 23A, 80-204 Gdańsk, Poland; marta.potrykus@gumed.edu.pl

**Keywords:** protease Lon, *Dickeya solani*, plant pathogen, virulence factors, motility, type III secretion system, pathogenicity, resistance to stress, *lon* expression, pectinolytic enzymes

## Abstract

The Lon protein is a protease implicated in the virulence of many pathogenic bacteria, including some plant pathogens. However, little is known about the role of Lon in bacteria from genus *Dickeya*. This group of bacteria includes important potato pathogens, with the most aggressive species, *D. solani*. To determine the importance of Lon for pathogenicity and response to stress conditions of bacteria, we constructed a *D. solani* Δ*lon* strain. The mutant bacteria showed increased sensitivity to certain stress conditions, in particular osmotic and high-temperature stresses. Furthermore, qPCR analysis showed an increased expression of the *lon* gene in *D. solani* under these conditions. The deletion of the *lon* gene resulted in decreased motility, lower activity of secreted pectinolytic enzymes and finally delayed onset of blackleg symptoms in the potato plants. In the Δ*lon* cells, the altered levels of several proteins, including virulence factors and proteins associated with virulence, were detected by means of Sequential Window Acquisition of All Theoretical Mass Spectra (SWATH-MS) analysis. These included components of the type III secretion system and proteins involved in bacterial motility. Our results indicate that Lon protease is important for *D. solani* to withstand stressful conditions and effectively invade the potato plant.

## 1. Introduction

Bacteria from the genus Dickeya, together with Pectobacterium, are classified as soft rot Pectobacteriaceae (SRP) [1]. They cause diseases of many economically important plants leading to significant financial losses all over the world [2,3]. *Dickeya solani* was first identified in 2005 and since then it has spread in Europe reducing yields of its primary host, potato [4,5]. *Dickeya solani* turned out to be a well-adapted and very successful pathogen, and due to its better adaptation in many regions, it has displaced another common potato pathogen*, Dickeya dianthicola*. Briefly, *D. solani* can infect the host plant with as little as 10 cells per tuber and has a broader temperature spectrum for infection compared to other SRP species [2]. SRP causes two types of plant diseases: blackleg and soft rot characterized by the blackening and wilting of a plant stem or tuber rot, respectively [5]. No effective methods to combat these pathogens have been developed so far [5]. The virulence factors that are primarily involved in the development of the disease symptoms caused by *D. solani* are plant cell wall degrading enzymes (PCWDE). This group of proteins encompasses pectinases, cellulases and proteases, whose joint action leads to maceration of plant tissues. Pectinases and cellulases are secreted via the type II secretion system (T2SS) and proteases are secreted via the type I secretion system (T1SS). However, an effective infection process requires also an array of other virulence determinants. These include the ability to actively move (motility) toward wounded tissue (chemotaxis), production of antioxidants, siderophores, secreted effectors and, finally, cellular factors that provide survival under unfavorable environmental conditions inside the host plant. *Dickeya* enters the plant via wounds or natural openings, and then it can penetrate the apoplast. There, the type III secretion system (T3SS), an essential virulence factor, is activated (reviewed in [6]). It consists of a pillus apparatus, so-called injectisome, connecting bacterial and plant cell cytoplasm [7] to directly inject effector proteins into the host. The effectors frequently manipulate host signaling pathways to disable the plant’s defense systems to enable successful infection (reviewed in [8]).

For proper functioning of the cell, it is necessary to maintain its homeostasis, which is especially problematic under stressful conditions. Pathogenic bacteria are frequently exposed to unfavorable conditions, both in the host and during transmission between the hosts. In their life cycle, *Dickeya* may encounter several types of stress. These include changes in temperature and pH, exposure to oxidants, as well as osmotically active compounds [6]. All the stressors mentioned above can cause protein damage. To maintain cellular proteostasis, a dedicated set of proteins, termed protein quality control system (PQCS), is employed. It includes proteases and chaperones, that, among others, protect the cell against the accumulation of harmful protein aggregates and participate in regulatory proteolysis [9,10]. Lon and ClpP are two major cytoplasmic proteases, responsible for 70–80% of the ATP-dependent proteolysis in the cytosol [11]. Lon degrades aberrant proteins, which can arise in the cell not only as a consequence of stress but also under physiological conditions. Additionally, native proteins with the degradation tags (in general nonpolar amino acids exposed to the solvent) can undergo proteolysis by the Lon protease. Lon regulates many important cellular processes through the degradation of relevant substrates. For example, in *Escherichia coli,* cell division and synthesis of capsular polysaccharide are regulated by proteolysis of the cell division inhibitor SulA and transcription regulator RcsA, respectively [12,13]. Moreover, in various bacteria, functions of Lon can be implicated in such processes as motility, DNA replication, sporulation and, finally, pathogenicity [14].

Lon is crucial for virulence of many animal and plant pathogenic bacteria [8,14]. The *lon* mutant of *Brucella abortus* displayed decreased pathogenicity in BALB/c mice, however only at an early stage of infection [15]. The more pronounced effect was observed in the case of *Salmonella enterica* serovar Typhimurium *lon* mutants, which were highly attenuated in mice [16]. *Pseudomonas aeruginosa* Lon protease is necessary for effective bacterial infection in the mouse acute lung and amoeba model [17]. *Agrobacterium tumefaciens* depleted of a functional *lon* gene was unable to induce tumors in *Kalanchoe diagremontiana* [18]. The *lon* mutants of *Pseudomonas syringae* pv. *phaseolicola* and *P. syringae* pv. *tomato*, in turn, showed reduced disease symptoms in bean and tomato models, respectively [19].

To determine the role of the Lon protein in *D. solani,* we constructed a *D. solani* Δ*lon* strain using a modified lambda red recombination protocol. This allowed us to provide an insight into the functions played by Lon in *D. solani* pathogenicity and growth under stressful conditions.

## 2. Results

### 2.1. Construction of the D. solani IPO 2222 Δlon and the Complemented D. solani IPO 2222 Δlon/lon Strains

**Figure 1:**
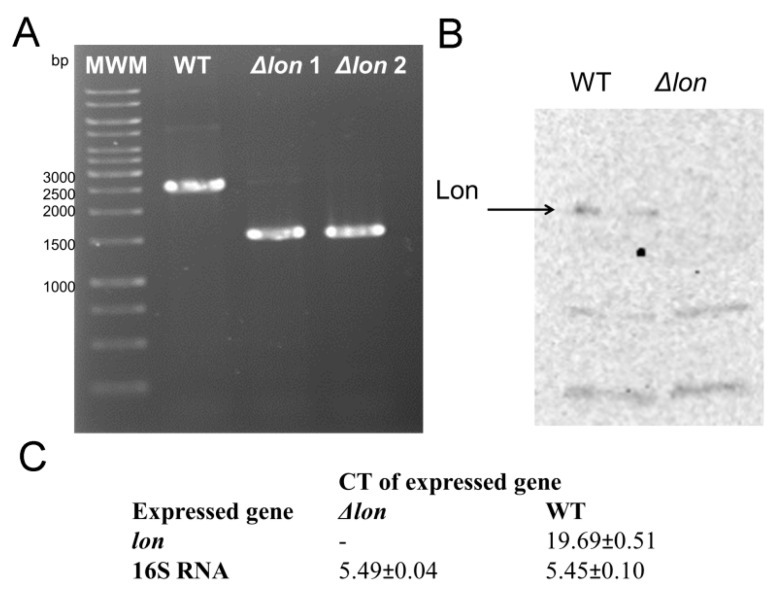
Confirmation of successful deletion of the *lon* gene in *D. solani* IPO 2222: (**A**) PCR analysis of genomic DNA isolated from the *D. solani* WT (wild type) and Δ*lon* mutant. The *lon* gene was replaced by a 1000 bp smaller kanamycin resistance gene. Δlon1 and Δlon2 denote two independent clones (**B**) immunodetection using the anti-Lon *E. coli* primary antibodies. (**C**) The qPCR analysis with the use of the *lon* gene-specific primers revealed that no cDNA amplification product was created within 50 cycles.

A *lon* deletion mutant of *D. solani* was constructed using the gene doctoring method, a modified protocol for lambda red recombination dedicated to the pathogenic bacterial strains. The gene encoding the Lon protease was substituted with the kanamycin resistance cassette, amplified from the pDOC-K plasmid. We confirmed the deletion of *lon* by three independent methods: (1) detection of the *lon* sequence on the *D. solani* chromosome by PCR using the primers complementary to the sequences flanking the *lon* gene; (2) detection of the *lon* transcript by real-time PCR; (3) immunodetection of the Lon protein using the anti-*E. coli* Lon rabbit antibodies. As can be seen in Figure 1, the *lon* gene and its product were not found in the strain *D. solani* IPO 2222 Δ*lon* but were present in the WT parental strain.

Single-copy complementation in the genome of the *D. solani* Δ*lon* strain was obtained using the *E. coli* MFD (Mu-free donor) *pir* conjugation strain. Immediately after the end of the *lon* gene, a marker gene encoding a pink Scarlett fluorescent protein was inserted. We confirmed the Δ*lon/lon* complementation at the gene and protein level (Figure S1).

**Figure 2:**
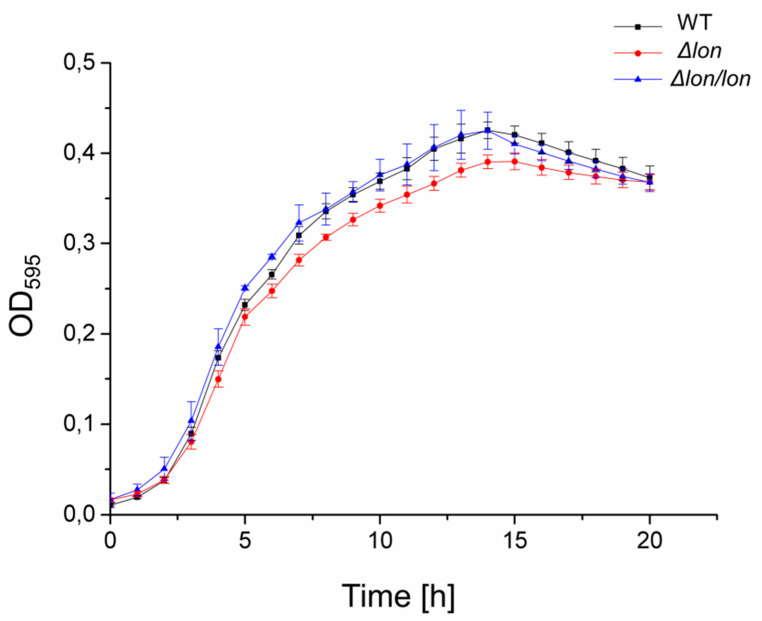
Growth of *D. solani Δlon.* Curves were determined with the use of a plate reader at 30 °C. OD_595_ (optical density (595 nm)) values in LB medium were averaged for four replicates.

A lack of the *lon* gene did not affect the growth of bacteria at the standard in vitro conditions (LB medium, 30 °C), as judged from the growth curves. The growth rates of the Δ*lon*, Δ*lon/lon* and WT *D. solani* cultures were comparable (Figure 2). Similar growth patterns of the Δ*lon* and WT strains have significantly facilitated the normalization of bacterial cultures in terms of age and cell density for subsequent stress sensitivity and pathogenicity tests.

### 2.2. The Expression of the lon Gene is Upregulated under Certain Stressful Conditions

Under stress, a cell activates a variety of defense mechanisms that are manifested by increased expression of the key protective proteins [20,21,22]. To check the importance of Lon in the stress response, we performed the qPCR analysis to measure levels of transcription of the *lon* gene. We chose the common stressors, possible to affect *D. solani* during saprophytic and pathogenic life cycle: elevated temperature, nonionic and ionic osmotica, acidic pH and oxidants [23].

**Figure 3:**
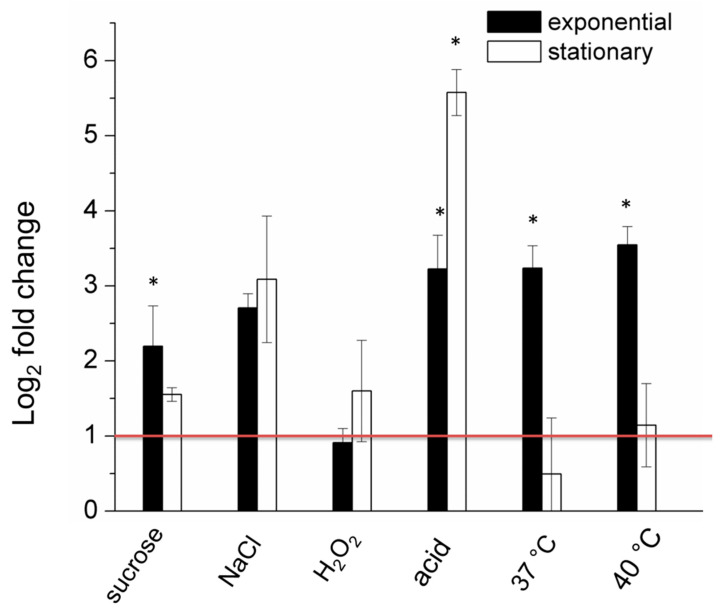
The relative log2 fold change of the expression levels of the *lon* gene in the *D. solani* cells under stressful conditions analyzed by qPCR. The data correspond to the means ± S.D. of three different samples, including three technical replicates. A red horizontal line indicates a relative two-fold increase in expression level. * indicates statistically significant (95% confidence interval) fold change in expression level according to the REST 2009 software.

We found that transcription of *lon* was significantly upregulated in the exponentially growing bacteria under stressful conditions such as hyper osmosis, acidic pH and high temperature (Figure 3). The most pronounced effect was exerted by acidic pH and elevated temperature (over three log_2_ fold increase). A milder effect was caused by the presence of a nonionic osmoticum, sucrose (over two log_2_ fold increase). In contrast, the expression of the *lon* gene did not change significantly in cells in the stationary phase of growth. The exception was the upregulation (over 5.5 log_2_ fold increase) of the *lon* gene expression in cells treated with acidic pH. Interestingly, the changes in the *lon* transcript level upon treatment with ionic osmoticum, NaCl, were pronounced regardless of the growth phase, although they were not statistically significant (Figure 3). In contrast, oxidative stress did not affect the transcription of *lon* (Figure 3). Hence, the Lon protease is rather not a component of the oxidation response in *D. solani.*

### 2.3. Lon Protease Plays a Protective Role under Ionic and High-Temperature Stresses

**Figure 4:**
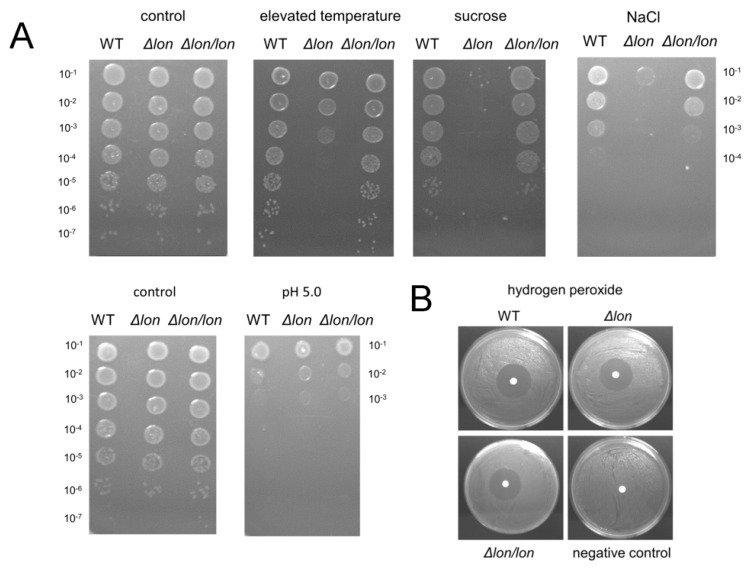
Growth of *D. solani* Δ*lon* under stressful conditions. (**A**) Overnight grown cultures were serially diluted and spotted on the LA (Luria Agar) agar plates, agar plates supplemented with 0.6 M sucrose or 0.3 M NaCl or on the LA medium adjusted to pH 5.0 when indicated. Bacteria grown on the LA agar plates at 30 °C refer to control. Disk diffusion assay with 1% hydrogen peroxide. As a negative control, sterile water was used (**B**). All plates were incubated at 30 °C except for the elevated temperature stress (39 °C).

Knowing that expression of *lon* is upregulated in cells in response to stress, we decided to check if the presence of the Lon protease in the cell is necessary for bacterial growth in the presence of selected stressors. Bacteria were exposed to the following stressful conditions: elevated temperature, ionic and nonionic osmotic shock, oxidative stress and low pH. We found that *D. solani* Δ*lon* was characterized by a decreased ability to form single colonies under three of five tested conditions. In particular, elevated temperature and presence of the nonionic osmoticum, sucrose, reduced viable cell counts by five and three orders of magnitude, respectively. Moreover, the Δ*lon* mutant colonies were very small under both tested conditions. The reintroduction of the *lon* gene into the *D. solani* Δ*lon* chromosome restored the wild-type phenotype of bacteria (Figure 4A). Hence, the strong phenotype of the mutant strain resulted from the lack of the Lon protease and not from putative additional suppressor mutations. The pronounced effect was also noticed under ionic osmotic stress: addition of NaCl resulted in a three-log reduction of cell counts of the Δ*lon* mutant with respect to the WT or complemented Δ*lon/lon* strain (Figure 4A). Acidic pH and oxidative stress affected all strains similarly (Figure 4A,B).

### 2.4. Deletion of the lon Gene Delays the Onset of the Infection Symptoms

**Figure 5:**
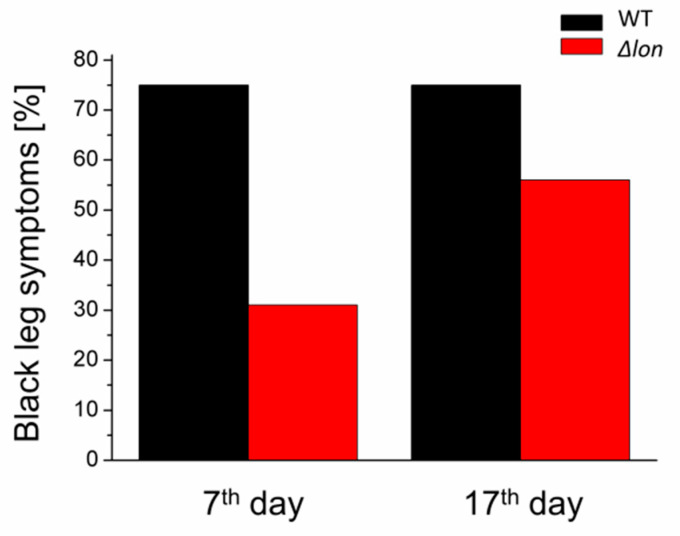
Pathogenicity of *D. solani* Δ*lon* in the whole potato plant model. Potato plants cv. Vineta were infected with WT and *lon* mutant strains. After 7 and 17 days of incubation at room temperature with a 16/8 photoperiod, the number of plants with blackleg symptoms was counted. Four plants watered with Ringer buffer represented a negative control. The number of infected plants with WT and Δ*lon* mutant was 16 for each strain.

To test the importance of the Lon protease for pathogenicity of *D. solani*, we performed in vivo infection of the potato plants under greenhouse conditions. This kind of experiment shows the ability of bacteria to invade plants through the root system and produce blackleg symptoms. Although the deletion of the *lon* gene did not significantly reduce the occurrence of disease, an obvious delay in the development of the disease symptoms was observed. On the seventh day postinfection, only 30% of the potato plants treated with the Δ*lon* mutant bacteria showed blackleg symptoms, compared to 75% of symptomatic plants infected with WT *D. solani*. On the 17th day, the differences were much less pronounced, with 55% and 75% of symptomatic plants infected with Δ*lon* and WT *D. solani*, respectively (Figure 5).

To evaluate the effects of the Δ*lon* mutation on the ability of bacteria to macerate plant tissues, we used three models: potato tubers and leaves of chicory and Chinese cabbage. In no case did we observe differences in the degree of tissue maceration (Figure S2).

### 2.5. The Deletion of lon Affects the Activity of Secreted Pectate Lyases

**Figure 6:**
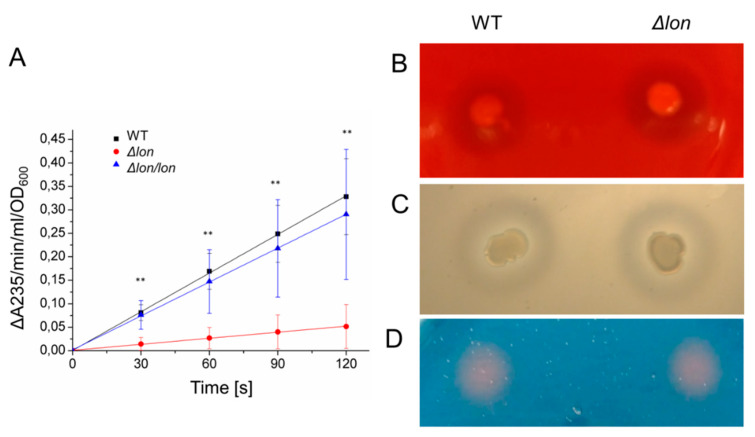
The activity of secreted virulence factors. (**A**) Pectinase activity was assayed as described in the Methods section with PGA as a substrate at 30 °C. ** *p* < 0.01 (*t*-test), *n* = 5. (**B**) Cellulases were assayed on M63Y with CMC. Seven microliters of bacterial cultures (10^8^ CFU/mL) were spotted on the medium and incubated for 72 h. Plates were stained with 2% Congo red solution. (**C**) To monitor protease activity, 7 µL of bacterial cultures (10^8^ CFU/mL) were spotted onto LA with skimmed milk and incubated for 48 h. (**D**) Siderophore activity was determined by spotting 10 µL of supernatant from overnight grown bacteria cultures onto chrome azurol S-agar plates. The picture was taken after 1 h of incubation at 30 °C. The experiments (**B**–**D**) were performed at least five times. The representative results are shown.

PCWDEs are the virulence factors that are directly responsible for the manifestation of disease symptoms. To check if altered pathogenicity of *D. solani* Δ*lon* results from changes in the level or activity of the enzymes that degrade the plant cell wall, we measured the activity of pectate lyases (major pectic enzymes), cellulases and proteases secreted from the mutant and WT *D. solani* cells. The enzymatic activity was assayed using PGA, modified cellulose CMC and casein, which are commonly used substrates for pectinases, cellulases and proteases, respectively. In the case of *D. solani Δlon,* the secreted pectate lyase activity was 85% lower than that of the WT strain (Figure 6A). However, the activities of the remaining hydrolytic enzymes were not affected by the *lon* mutation (Figure 6B,C). The level of other secreted virulence factors, siderophores, also remained unchanged (Figure 6D).

### 2.6. Lon Protease is Essential for Efficient Motility

**Figure 7:**
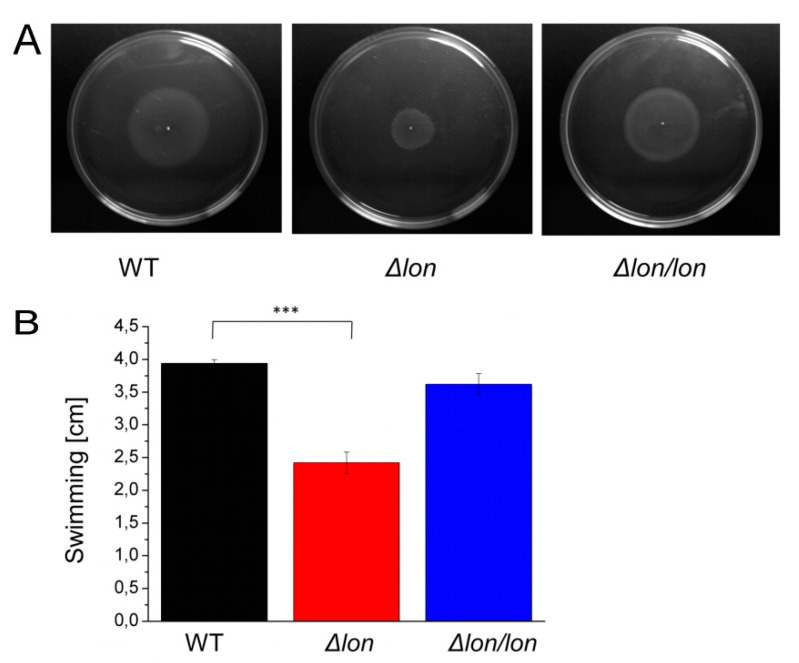
Swarming and swimming motility of *D. solani* Δ*lon*. (**A**) Representative pictures of bacteria swarming on 0.8% TSA after 12 h incubation at 30 °C. The experiments were performed at least five times. (**B**) Swimming was examined in a 0.3% MMA medium for 24 h at 30 °C. The diameter of the bacteria spreading area was measured. Presented data represent values for 5 biological replicates. *** *p* < 0.001 level (*t*-test).

Motility is one of the key factors for a successful invasion of the plant host. To determine if the observed delay of the blackleg symptoms development in the potato plants infected by *D. solani* Δ*lon* can be associated with altered bacterial motility, we examined two types of motility, swimming and swarming. While both types rely on the rotation of flagella, swimming is characteristic for an individual cell and is enhanced by chemotaxis. In contrast, swarming is common for a group of bacteria [24]. Indeed, the lack of Lon resulted in the altered motile phenotype of bacteria. The mutated strain showed considerable reduction in the swarm (Figure 7A) and 30% reduced swimming motility in the presence of galactose as a chemotactic agent (Figure 7B).

### 2.7. Comparison of Proteomic Profiles of the D. solani Δlon and WT Cells under Physiological and Stressful Conditions

To gain more detailed insight into the properties of the Δ*lon* mutant cells, we compared the proteomes of the mutant and WT strains under physiological, as well as stress conditions, by the means of SWATH-MS (Sequential Window Acquisition of All Theoretical Mass Spectra) analysis. SWATH-MS is an advanced analysis method of proteomic data, recommended for quantification of identified peptides. It allows quantitative comparison of protein levels between different species or treatments due to the construction of a peptide spectral library [25].

It is well known that treatment with severe stressful agents can cause abnormal changes in the level of individual macromolecules [20] so we decided to subject the *lon* mutant to a rather mild stress—a short incubation at 40 °C.

**Figure 8:**
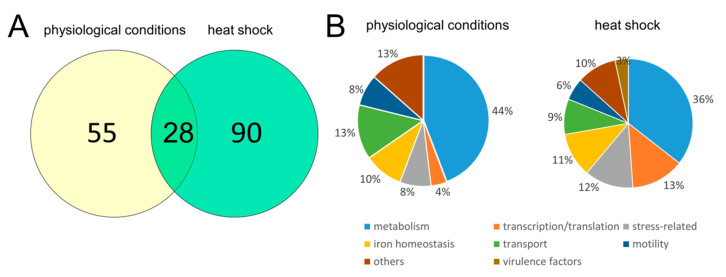
Number (**A**) and percentage (**B**) of proteins whose expression level was changed in the Δ*lon* mutant under physiological and heat shock conditions. Data are categorized into 8 groups depending on the protein function. Physiological and heat shock conditions refer to temperatures 30 °C and 40 °C, respectively.

The analysis identified a total of 635 proteins, for which at least two peptides per protein were quantified (Table S1). Deletion of the *lon* gene induced global changes in the *D. solani* proteome. We narrowed the number of differentially expressed proteins by applying the following cut-off criteria: *p* < 0.05, as well as fold changes below 0.5 or above 2.0. This resulted in 38 proteins with altered abundance in Δ*lon* compared to WT under physiological conditions and 60 proteins under stress conditions. Hence, the changes in the mutant proteome were more pronounced following the temperature shift than under control conditions, which may reflect the increased need for the Lon function during stress. In particular, the deletion of the *lon* gene resulted in upregulation of 17 or 41 proteins and downregulation of 13 or 19 proteins under physiological or stress conditions, respectively. Of these, 28 proteins shared a similar pattern of expression under both tested conditions (Figure 8A).

We grouped differentially expressed proteins into eight categories, depending on their physiological functions (Table 1). These include involvement in motility, iron metabolism, stress response, transport, general metabolism, transcription/translation, virulence and others. Percentage of proteins representing particular groups differs between control and induced conditions, however, the most abundant class encompasses proteins associated with general cell metabolism (Figure 8B). Consistent data were obtained for proteins involved in bacterial motility, namely all of them were repressed in Δ*lon* compared to WT. Among them, we identified flagellin, a structural component of bacterial flagella, and proteins responsible for chemotaxis (CheW, a positive regulator of CheA protein activity and CheA, signal transduction histidine kinase CheA). On the contrary, deletion of *lon* caused an increase in the cellular content of a group of proteins associated with virulence. They are all engaged in the T3SS and include hairpins, HrpN, a homolog of HrpW (Various polyols ABC transporter, permease component 2), as well as HrpA, Hrp pili protein. Deletion of *lon* differentially affected levels of proteins related to iron metabolism. We observed the upregulation of the proteins involved in the Fe-S cluster assembly (ISCU) and biosynthesis of achromobactin siderophore. However, the level of proteins involved in the synthesis of another siderophore, enterobactin, was decreased (for example isochorismate synthase enterobactin siderophore). The mutant strain was characterized by an increased content of several proteins engaged in transcription and translation. Among them, we could distinguish ribosomal proteins (50S ribosomal protein L27 and L7/L12), transcription factors (CytR, DksA) and RNA-binding protein Hfq. We obtained a similar trend regarding stress-related proteins, like ClpP protease and cold shock response proteins (CspE and CspG). A total of 77% of proteins associated with transport activity were downregulated, including histidine ABC transporter and efflux pump membrane transporter. The group named “others” comprises uncharacterized proteins or polypeptides which were not assigned to any other category. Among them, we identified putative membrane protein A0A2K8W3L1_9GAMM whose expression was increased more than 100-fold under both tested conditions. Protein blast indicated very close homology to periplasmic ComEA from many bacterial species, with the closest homology (100% coverage, 99% identity) to *Dickeya fangzhongdai*. ComEA is essential for DNA uptake in naturally competent bacteria, like *Bacillus subtilis* [26]. In Δ*lon*, we also observed an increased cellular level of S-ribosylhomocysteine lyase, which is associated with quorum sensing.

## 3. Discussion

For successful infection, a pathogen must have the capability to enter the host, overcome the host defense systems, acquire nutrients, multiply and disseminate. All these stages are associated with constant exposure to a variety of potentially harmful conditions, both in and outside the host. Hence, successful pathogens should have well-developed virulence mechanisms but also efficient stress response systems. Proteolytic enzymes were shown to play numerous crucial roles in bacterial virulence. They can directly act as virulence factors, but also can contribute to virulence by regulating the production of virulence factors and/or as components of protein quality control systems to provide cellular proteostasis. One of the latter cases is the Lon protease which is indispensable for stress tolerance and virulence of many bacterial species causing infectious diseases.

Our work showed that the Lon protease is necessary for the bacterium *D. solani* to resist exposure to stress, including ionic- and nonionic osmotic stress, as well as high temperature. This finding is in agreement with data obtained for other bacterial species. Lon has a well-documented role in bacterial viability under heat and salt stress [14]. In *E. coli*, expression of the *lon* gene depends on sigma32 (RpoH) transcription factor [27,28], activated under heat shock and osmotic stress [29,30]. High temperature stimulates expression of *lon* in *E. coli* [31] and *Francisella tularensis* LSV [32]. Ionic osmotic stress is responsible for the elevated level of *lon* expression in *B. subtilis* and *Dickeya dadantii* [33]. Consistently, in *D. solani*, *lon* expression was significantly elevated in exponentially growing bacteria following exposure to elevated temperature; a positive trend was also observed in case of salt stress. Heat shock is more harmful to bacteria in the logarithmic than the stationary growth phase [34], which may explain stronger stimulation of the *lon* gene in the logarithmically growing cells. Higher demand for Lon, suggested by the upregulation of *lon* under certain stress conditions, can explain reduced growth of the Δ*lon* bacteria under thermal and osmotic stress. Additionally, the elevated level of stress-related proteins in proteomes of bacteria treated with 40 °C, revealed by the SWATH-MS analysis, indicates the higher stress level in the mutant cells than the WT. Increased expression of RecA and protease HtpX suggests a higher frequency of DNA damage and probably impaired integration of membrane proteins, respectively, according to data published for *E. coli* [35,36]. Finally, the increase in the abundance of the second important cytosolic protease, ClpP, in the deletion strain, reveals the essential role of Lon protease in the quality control proteolysis in the cytoplasm. Most probably, ClpP takes over some of the Lon functions. However, it should be noted that the Δ*lon* strain showed a temperature-sensitive (TS) phenotype, so ClpP cannot substitute for Lon under heat shock conditions.

To our surprise, the Δ*lon* mutants were particularly vulnerable to treatment with sucrose. Nonionic osmotic agents, like sucrose, are considered less harmful for a cell than the ionic ones, (e.g., NaCl) [37]. We did not find data regarding the relationship between Lon and the response to osmotic stress caused by high sucrose in any bacterial species. Hence, this important function of Lon in resistance to nonionic osmotic stress needs to be elucidated. Although the expression of *lon* was strongly upregulated in the response of *D. solani* to low pH, we did not observe differences of growth between the mutant and WT strains. Possibly, the ClpP protease or other component of the protein quality control system takes over the duties of Lon under this type of stress. The involvement of the Lon protease in resistance to acidic stress is rather poorly investigated across different bacterial species. In *E. coli,* Lon is responsible for the degradation of the activator of acidic resistance, GadE, playing a role in the termination of the stress response [38]. Additionally, *S. enterica* serovar Typhimurium requires this protease to successfully cope with low pH [16]; however, the precise mechanism was not provided. Interestingly, the closely related species, *D. dadantii*, showed repression of *lon* expression in the low pH medium (although not statistically significant) [33], which is opposite to our findings. This may reflect interspecies differences but also certain differences in experimental design. As with *D. dadantii* [33], in *D. solani* the expression of *lon* was not affected by oxidative stress-induced with hydrogen peroxide. Hence, in Dickeya, the oxidation response most probably involves other components of PQCS.

Production of functional virulence factors is frequently dependent on specific proteolytic activity in the cell [39,40]. To verify the involvement of Lon in *D. solani* virulence, we checked the activity of the most abundant secreted virulence factors. We found that activity of the extracellular pectinases was reduced in the case of Δ*lon* mutant. Pectinases constitute a heterogeneous group of proteins. They differ in substrate specificity, abundance and role in virulence but all are secreted via T2SS [41]. At least 10 pectinases produced by *D. solani* have been identified so far [41]. The commonly used tests (including the one used in this work) measure a total pectinase activity and do not allow to distinguish between the individual pectinases. Analysis of the Δ*lon* and WT *D. solani* proteomes did not reveal differences in the cellular content of pectinases. However, we do not know if these proteins were efficiently transported outside the cell. As this is the first report of the function of Lon in the soft rot bacteria, there is no information regarding the relationship between Lon and PCWDE. No literature data is indicating the possibility of regulating the T2SS transport system by Lon. Moreover, the activity of extracellular cellulases, also T2SS dependent, remained unchanged in the *lon* mutant. Hence, the involvement of Lon in the regulation of T2SS is unlikely. Thus, further research is needed to clarify the Lon-dependent protease regulation of the secreted pectinase activity.

We did not observe any changes in the production of siderophores, although the lack of Lon protease lowered the abundance of several proteins engaged in iron metabolism. A higher level of proteins with function in iron-sulfur (Fe-S) protein biogenesis (IscU, ErpA) in the deletion strain may indicate them as potential substrates for the Lon protease. That is true in *Saccharomyces cerevisiae,* where a Lon homolog, Pim1, degrades Isu, a homolog of IscU [42]. The increase in the amount of the negative transcriptional regulator Fur is very interesting. Whether it is degraded by Lon is not known and no such Lon function was found in other bacteria. Almost a 10-fold increase of the Fur level in Δ*lon* may explain the decreased amount of certain Fur-dependent proteins involved in the synthesis of siderophores (e.g., enterobactin synthetase component F in *E. coli* [43]. On the other hand, the other enzymes from the siderophore biogenesis pathway were upregulated (like achromobactin biosynthesis protein AcsD), presumably compensating for the downregulated components to maintain iron homeostasis in the mutant cells.

A lack of Lon exerted a significant impact on the mobility of *D. solani.* We demonstrated that the cells deprived of Lon showed impaired swarming and swimming motility. This can be explained by the reduced levels of flagellin and positive regulators of chemotaxis in *D. solani* Δ*lon*, as revealed by the proteomic analysis. Depending on the bacterial species, the effects of the *lon* mutations on bacterial motility may be radically different. On one hand, the *lon* mutation can cause stimulation of motility. Good examples are *Proteus mirabilis* and *B. subtili*s, in which *lon* mutants showed better swarming [44,45]. In these bacteria, Lon degrades master activators of flagellin biogenesis- FlhD and SwrA, respectively. Hence, in the *lon* backgrounds, these activators became stabilized, leading to a hypermotile phenotype. In contrast, the *lon* mutant of *Erwinia amylovora* was characterized by a nonswarming phenotype [46]. In *E. amylovora,* a mutation in the *lon* gene resulted in the accumulation of RcsA/RcsB that negatively regulates transcription of *flhD*, the master regulator of flagellar biosynthesis. Finally, a lack of Lon may not affect bacterial motility at all, as shown for *S. entrica* serovar Typhimuirum [47]. The results obtained in this work suggest an indirect role of Lon in the motility of *D. solani*, analogous to that of *E. amylovora* Lon, as the *flhD* gene is present in *D. solani* genome and the *lon* mutant was characterized by a decreased flagellin content. Interestingly, *D. solani* Δ*lon* was characterized by the two-fold increased level of the CytR transcription factor, which in *Pectobacterium carotovorum* positively stimulates genes associated with motility: *fldH, fliA, fliC* and *motA* [48]. However, the increased content of the CytR protein in *D. solani* Δ*lon* obviously was not sufficient to compensate for the other Δ*lon* -dependent effects that lead to a reduced flagellin and chemotaxis protein levels, or CytR is not involved in regulation of motility in *D. solani*.

The phenotypes of *D. solani* Δ*lon* reported in this work suggested that the presence of Lon may be necessary for efficient infection of the potato plant. Indeed, we found that the process of development of blackleg symptoms in the plants infected with the mutant strain was markedly delayed in respect to infection with the WT *D. solani.* On the other hand, Lon was not essential for the maceration of plant tissues in vitro. Both types of infection tests differ fundamentally in terms of the availability of plant tissues for bacteria. In the whole plant model, the bacteria were placed in the soil, from where they must have got into the wounded tissue, in this case, roots. In this context, the motility and chemotaxis toward chemical signals (e.g., jasmonic acid) are crucial. Consequently, the nonmotile mutant strains are characterized by a lack or reduced virulence, as they may encounter severe problems with entering and/or spreading in the host [49]. In the slice or leaf model, bacteria were spotted directly into the wounded tissue, so chemotaxis and motility were less important. In the tissue model, the basis of infection’s success lies in the production of PCWDE, iron homeostasis, and bacterial fitness under pH, oxidative and osmotic stresses (reviewed in [6]). As we did not observe any difference in the degree of maceration of the tuber or leaf tissues between the WT and mutant strains, we assumed that Lon was not essential for bacterial survival in the plant under experimental conditions. The secreted pectinase activity of the Δ*lon* mutant was reduced but was still enough for efficient plant maceration. Hence, we concluded that the observed delay in the potato plant infection process was most probably due to reduced motility of the Δ*lon* strain.

Lon is known to be engaged in the regulation of T3SS, however, the particular effects of *lon* mutations are species-dependent. In *P. aeruginosa* and *Yersinia*, the deletion of the *lon* gene results in the downregulation of T3SS [17,50]. However, the opposite effect was demonstrated in *E. amylovora* and *P. syringae* [46,51]. We found that in *D. solani,* Lon negatively affected the level of proteins associated with the type III secretion system: HrpN and a homolog of HrpW as well as HrpA (structural protein of T3SS pillus). These results are consistent with data obtained for *E. amylovora* and *P. syringae*. In these bacteria, Lon indirectly downregulates transcription of *hrpL* gene coding for HrpL, the RNA polymerase sigma factor, which is necessary for the initiation of transcription of T3SS genes. In *P. syringae* and *E. amylovora* this is mediated via degradation of the transcriptional activators of the *hrpL* gene, HrpR and HrpS, respectively [46,51]. In addition, Lon also indirectly regulates HrpS levels through RcsA proteolysis in the *E. amylovora* cells. RcsA is a component of RcsA/RcsB regulatory complex, which activates transcription of the *hrpS* gene [46]. Finally, Lon of *E. amylovora* degrades HrpA [46], which may also be true in the case of *D. solani*, as we observed an elevated level of this protein in the mutant strain. Interestingly, in *E. coli*, the Lon substrates, CspG and CspE proteins, positively regulate the *rcsA* expression [52]. In the *D. solani Δ lon* strain, CspG and CspE were upregulated. It cannot be ruled out that they can also be degraded by the Lon protease, which would additionally suppress the expression of T3SS.

In addition to the IscU, HrpA and CspG/E proteins discussed above, the proteome analysis revealed one more potential substrate for the *D. solani* Lon protease. We observed elevated levels of the RNA-binding protein Hfq, which is a known substrate for Lon in *P. aeruginosa* [53]. Fernandez and colleagues [53] suggested that the accumulation of Hfq can contribute to reduced motility of the *lon* strain. In the case of *D. solani,* these findings need verification.

The most pronounced effect of the *lon* deletion was observed in the case of the protein A0A2K8W3L1_9GAMM, a homolog of ComEA, whose level was more than 100-fold higher in Δ*lon*. ComEA is necessary for natural cell competence. However, *D. solani* was not reported to exhibit natural competence. Moreover, the calcium chloride transformation method of *D. solani* is highly inefficient and *D. solani* spp. genomes lack in general large plasmids [54]. Interestingly, in *Vibrio cholerae comEA* expression is activated by the transcriptional regulator CytR [55]. We also observed an increased level of CytR in the deletion strain, which may be the reason for the upregulation of the ComEA protein. However, the relationship between CytR and Lon protease requires further investigation.

Finally, SWATH-MS analysis revealed that the deletion of *lon* affected the balance among proteins involved in cellular metabolism and transport. This is not surprising as the housekeeping proteases, in general, regulate metabolic activities [56] and this is also true for Lon [14].

In light of the data presented in this work, the Lon protease is a protein that plays very important roles in *D. solani* physiology, both under physiological and stressful conditions. Lon was shown to be required for the full virulence of *D. solani* in the whole plant model. Lower pathogenicity of the Δ*lon* bacteria may result from impaired expression/activity of certain virulence factors, including motility and secreted pectinases, but also from decreased ability to withstand stressful conditions. To our knowledge, this is the first report that describes the function of the Lon protein in the bacterial species from the SRP group.

## 4. Materials and Methods

### 4.1. Materials

Restriction enzymes and dNTPs were purchased from EURx (EURx Sp. z o.o., Gdańsk, r, Poland); PrimeSTAR GXL polymerase for construction of deletion strain from Takara Bio Inc. (Shiga, Japan); T4 DNA ligase, T5 Exonuclease and Phusion High-Fidelity polymerase from New England Biolabs (USA) and reagents for media and buffers from Sigma-Aldrich (Saint-Louis, MI, USA), and Chempur (Piekary Śląskie, Poland). Oligonucleotides were synthesized by Eurofines Scientific (Luxembourg, Luxembourg) or Sigma-Aldrich (Saint Louis, MI, USA).

### 4.2. Bacterial Growth Conditions

Bacterial strains and plasmids used in this study are listed in Table 2. Bacteria were grown in the minimal medium M63Y (0.1 M KH_2_PO_4_, 15 mM (NH_4_)_2_SO_4_, 9 µM FeSO_4_, 1 mM MgSO_4_, 1 mg/L vitamin B1 and 0.3% glycerol, pH = 7.0) [57], LB broth (1% tryptone, 0.5% yeast extract, 1% NaCl) or SOC (2% tryptone, 0.5% yeast extract, 10 mM NaCl, 20 mM glucose, 2.5 mM KCl, 10 mM MgSO_4_) with shaking at 30 °C, unless indicated otherwise. For all analyses, overnight cultures were diluted 1:50 with M63Y or LB and cultured for the next 16 h until they reached the early stationary growth phase. Then, they were used in experiments or diluted again 1:50 with M63Y and grown for 4.5 h to reach a midexponential phase. The overnight *D. solani Δ lon* cultures were grown in the medium supplemented with kanamycin (0.1 mM); the cultures directly subjected to experiments were devoid of the antibiotic to provide comparable growth conditions of all bacterial strains.

Growth curves were determined with the use of the EnSpire plate reader (PerkinElmer, Waltham, USA) in a 24-well nontreated plate (#702011 Wuxi NEST Biotechnology Co., Ltd., Wuxi, MA, China). Overnight grown cultures were diluted 1:50 with LB medium to a final volume of 1 mL. Bacteria were grown with orbital shaking (120 rpm) and OD_595_ measurements were taken every hour. Growth was monitored for 20 h at 30 °C. The final OD values were averaged for four biological replicates.

To induce stress, 4 µL aliquots of 10-fold serial dilutions of the stationary bacterial cultures in Ringer buffer (147 mM NaCl, 4 mM KCl, 2.24 mM CaCl_2_ × 2H_2_O) were spotted onto the LA agar plates (LB broth with 1.5% agar, control conditions and temperature stress), LA agar plates supplemented with 0.6 M sucrose (nonionic osmotic stress) or 0.3 M NaCl (ionic osmotic stress), or adjusted with malic acid to pH 5.0 and incubated for 20–48 h at 30 °C or 39 °C (heat shock). The Agar disk diffusion method was used to study the susceptibility of bacteria to oxidative stress. One-hundred microliters of overnight culture diluted 100 times was spread onto the LA medium. A sterile disk (6mm) of Whatman 1M paper was placed on an LA plate and then 8 µL of 1% hydrogen peroxide solution was spotted on it. The water-soaked disk served as a negative control. Plates were incubated at 30 °C for 24 h.

To analyze gene expression, the WT strain grown in M63Y to the midexponential or early stationary phase was subjected to the selected stress conditions for 15 min [33]. Briefly, NaCl and sucrose were added to the cultures to a final concentration of 0.3 M and 0.32 M, respectively. The shift of temperature was obtained by incubation of bacteria for 15 min in a water bath at 37 °C or 40 °C with shaking. As a control, bacteria grown in the absence of a stressor at 30 °C were used. To stabilize mRNA, a cold solution of 5% acid phenol (BioShop Canada Inc., Ontario, Canada), in 99.9% ethanol was added to the bacterial culture at the 1:9 ratio and bacteria were immediately put on ice.

### 4.3. Construction of the lon Deletion Strain

The deletion of the *lon* gene from the *D. solani* chromosome was performed according to gene doctoring protocol [62]. Briefly, primers with homology to the upstream/ downstream regions of the kanamycin resistance cassette from pDOC-K plasmid and 40 bp flanking region of the *lon* gene were designed (Table 3). Restriction sites for XhoI and KpnI were added at 5′ end of lonkan L and lonkan R primers, respectively. PCR reaction was performed with pDOC-K as a template. The PCR product and pDOC-C plasmid were digested with XhoI and KpnI restriction enzymes, then the PCR product was cloned into the backbone of pDOC-C, generating pDFDOC-C-lon. Plasmid pABSCE and pDFDOC-C-lon were electroporated into *D. solani* cells and the transformants were selected for resistance to chloramphenicol and ampicillin. The proper recombinants were selected based on a lack of ability to grow on a medium with 8% filtrated sucrose, as the pDFDOC-C-lon plasmid contains the *sacB* gene. To check this, 1 mL portions of LB medium with 0.5% glucose and appropriate antibiotics were inoculated with single colonies and incubated with shaking at 30 °C for 4 h. The culture was centrifuged (1167× *g*, 2 min), pellet resuspended in 1 mL LB medium supplemented with 0.1–2% arabinose and incubated at 30 °C with shaking until turbid. Bacteria were spotted on the LA plates supplemented with 8% sucrose (sterilized by filtration) and kanamycin and in parallel on LA with kanamycin. Next, the colonies that did not grow on the sucrose plates were tested for pDFDOC-C-lon and pABSCE plasmid loss by the selection of bacteria unable to grow on ampicillin and chloramphenicol. PCR with primers homologous to the flanking region of a *lon* gene (Table 3) was performed to confirm the deletion of the *lon* gene.

### 4.4. Single-Copy Complementation

Complementation strain was obtained by reintroduction of the WT *lon* gene into its native locus on the chromosome in the *D. solani* Δ*lon* cells using conjugation strain *E. coli* MFD *pir*, according to [60]. To do this, a plasmid containing the WT *lon* gene with the *mScarlet* gene coding for a fluorescent pink protein as a marker was obtained by the Gibson assembly approach. Briefly, four insert fragments were amplified (Table 3) and mixed with the allelic exchange pRE112 vector cut with the SmaI restriction enzyme, and reaction mix [65]. pRE112 carries the *sacB* marker gene and chloramphenicol resistance gene. The total amount of DNA in the reaction was 150 ng.

The resulting reaction product was transformed into *E. Coli* DH5α *pir* and the subsequently isolated plasmid was named pLonScar. The pLonScar plasmid was introduced into *E. coli* MFD *pir* in the presence of 0.3 mM diaminopimelic acid (DAP, Sigma-Aldrich, Saint Louis, MI, USA) in the medium to allow the growth of bacteria [60]. The overnight cultures of *E. coli* MFD *pir* (pLonScar) and *D. solani* Δ*lon* were mixed in a 3:1 ratio (total volume 800 µL) and centrifuged (1677 ×*g*, 2 min). The pellet was suspended in 30 µL of LB and spotted on an acetate cellulose filter placed on the LA solid medium supplemented with chloramphenicol but without DAP to eliminate *E. Coli* MFD *pir*. After 24 h incubation at 30 °C, bacteria were recovered by shaking the filter in 1 mL M63Y. Bacteria were spotted onto the LA agar plates with chloramphenicol. Then, individual colonies were tested for loss of the pRE112 plasmid. Briefly, cultures in the middle logarithmic growth phase were serially diluted and spread on LA without NaCl but supplemented with 10% of 0.22 µL filtered sucrose. Only cultures unable to grow on medium with sucrose were subjected for further verification. Ultimately, pink colonies sensitive to chloramphenicol and kanamycin were considered as true recombinants.

### 4.5. Plasmid and Genomic DNA Purification

Genomic and plasmid DNA were isolated using Genomic Mini (A&A Biotechnology, Gdynia, Poland) and Plasmid Mini (A&A Biotechnology, Poland) kits, respectively, according to the manufacturer’s protocols.

### 4.6. Preparation of Electrocompetent Cells and Electroporation

50 mL of SOC medium was inoculated with an overnight culture of *D. solani* at a 1:50 ratio. Bacteria were grown until OD_595_ of 0.45–0.5. The culture was centrifuged (7 min, 5063× *g*, 20 °C), pellet resuspended in 50 mL of deionized water, mixed thoroughly and forwarded to centrifugation (8 min, 5872× *g*, 20 °C). The cells were suspended in 25 mL of deionized water, mixed and centrifuged again (as above). The supernatant was precisely discarded, bacteria suspended in 1 mL of deionized water and split into 60 µL portions. Then, the cells were immediately used for electroporation [66]. Briefly, up to 50 ng of DNA was mixed with electrocompetent cells and transferred to 0.1 cm gap electroporation cuvettes (room temperature) for electroporation (1.25 kV). The bacterial suspension was diluted with 1 mL of SOC medium and incubated for up to 3 h for recovery. The 100 µL aliquots and the remaining bacteria (after 1 min 1677× *g* centrifugation and resuspension in 100 µL SOC) were plated onto the LA solid medium supplemented with an appropriate antibiotic and incubated for 24–36 h at 30 °C.

### 4.7. In Vivo Infection of the Potato Plants

The pot grown potato plants were obtained from sprouts. Briefly, the potato tubers of cultivar Vineta, obtained locally in Gdańsk, Poland, were stored in the dark until the development of sprouts (app. 3–4 months). Sprouts of a length of ca. 5 cm were carefully removed from tubers, planted into 7 cm square pots with potting soil (COMPO SANA ca 50% less weight) and placed on the windowsill for rooting and shoot development. After approximately two weeks, the rooted green plants were transferred to the humid growth chamber and grown under the white fluorescent light (48 × 5 W, Mars Hydro Reflector 48 with 16:8 h light: dark photoperiod). The potato plants at least 10 cm high were subjected to pathogenicity tests. Four overnight cultures of WT and four of *D. solani* Δ*lon* grown in LB medium were diluted with Ringer buffer to OD_595_ ~0.125, corresponding to 10^8^ CFU/mL. The roots of each potato plant were wounded with the scalpel about 2 cm from the stem. Plants were watered with 30 mL of bacterial suspensions and left for an hour. Then, the filtrate was discarded. Each bacterial culture was used to infect 4 plants. As a negative control, four plants treated with Ringer buffer were used. The experiment was carried out for 17 days and the percentage of plants with blackleg symptoms was estimated.

### 4.8. Pathogenicity on Potato Tubers, Chicory and Chinese Cabbage Leaves.

CFU/mL of each overnight bacterial culture was normalized to 10^8^ with Ringer buffer and then 10-fold serially diluted. Potato tubers were sterilized with a 10% bleach solution for 20 min, then submitted to three washes with sterile deionized water for 20 min each. The tubers were cut into 1 cm thick potato slices; in each slice, a little hole was pierced with a sterile pipette tip. Chicory and Chinese cabbage leaves were washed with sterilized deionized water and incised with a sterile scalpel. Ten-microliter aliquots of bacterial culture of 10^7^ CFU/mL were spotted onto the plant material. Ringer buffer was used as a negative control. The infection assays were performed in the humid boxes, at 30 °C for up to two days: one day for Chinese cabbage, two days for chicory leaves and for potato slices.

### 4.9. Determination of Motility

For swimming, a single bacterial colony (five replicates per strain) was inoculated into the semisolid agar plate with 0.3% MMA medium (40 mM K_2_HPO_4_, 22 mM KH_2_PO_4_, 0.41 mM MgSO_4_ × 7H_2_O, 0.3% agar) supplemented with 1 mM galactose. The plates were incubated under aerobic conditions at 30 °C for 48 h. The diameter of the bacterial spreading area was measured.

To monitor swarming motility, a single bacterial colony (five replicates per strain) was inoculated into the plate with 0.5% TSA (tryptone soy broth) medium (Oxoid, Basingstoke, UK) supplemented with 0.5% agar). Plates were incubated under aerobic conditions at 30 °C for 12 h. Both tests were repeated two times.

### 4.10. Determination of Secreted PCWDE Activity

The measurement of pectinolytic activity was performed as described in [67]. Briefly, bacteria were cultured in the M63Y medium until an early stationary phase and centrifuged (13,148× *g*, 2 min). Then, 260 µL aliquots of supernatant were diluted with equal volumes of distilled water. Briefly, 500 µL samples of the diluted supernatant were mixed with 1.5 mL of PGA (polygalacturonic acid, Sigma-Aldrich, Saint Louis, MI, USA) buffer (100 mM Tris–HCl (pH 8.5), 0.35 mM CaCl_2_ and 0.24% sodium polygalacturonate) warmed up to 30 °C. The reaction consisting in the formation of unsaturated products from polygalacturonate [68] was monitored spectrophotometrically by measurement of increase of absorbance at 232 nm for 2 min, every 30 s. Absorbance values obtained for PGA buffer were subtracted from values obtained for unsaturated product. The spectrophotometer was calibrated with distilled water. Pectynolytic activity was presented as ΔA235/min/mL/OD_595_. The experiment was repeated two times for each strain with at least three replicates.

The extracellular cellulase activity was assayed as described in [69]. Briefly, bacteria grown in the M63Y medium to the stationary phase were diluted with Ringer buffer to 10^8^ CFU/mL. Seven-microliter aliquots of bacterial cultures were spotted on the agar plates with carboxymethyl cellulose CMC (M63Y medium supplemented with 1.5% agar and 1% CMC). The plates were incubated at 30 °C for 48 h and then subjected to staining with 2% Congo red solution for 20 min. The diameters of the arisen halo were measured. The experiment was repeated two times for each strain with five replicates.

To measure the extracellular protease activity, bacteria cultivated in the M63Y medium to the stationary phase were diluted with Ringer buffer to 10^8^ CFU/mL. Seven-microliter aliquots of bacterial cultures were spotted on the milk agar plates (the LA medium supplemented with 5% skimmed milk). Plates were incubated at 30 °C for 48 h and the diameters of the arisen halo were measured. Each strain was tested two times with five replicates.

### 4.11. Siderophore Activity Assay

Ten-microliter aliquots of supernatants from the stationary cultures (grown in M63Y medium for 16 h) were spotted on the chrome azurol S-agar plates [70]. To prepare the chrome azurol S-agar medium the following solutions were prepared: (1) main medium, (2) 10% deferrated casamino acids (CAS), (3) 0.1 M CaCl_2_, (4) filtered 1 mM FeCl_3_ × 6H_2_O in 10 mM HCl, (5) CTAB (cetrimonium bromide, Sigma-Aldrich, Saint Louis, MI, USA). To prepare the main medium solution the components, KH_2_PO_4_ (3 g), NaCl (0.5 g), NH_4_Cl (1.0 g), MgSO_4_ × 7H_2_O (0.2 g), sucrose (4.0 g) and agar (15.0 g), were dissolved in 850 mL of 0.5 M Tris–HCl, pH 6.8 and sterilized. The deferrated casamino acids were prepared by the removal of ferrous ions with 3% 8-hydroxyquinoline in chloroform. The sterilized main solution was supplemented by 30 mL of 10% deferrated casamino acids, 10 mL of 0.1 M CaCl_2_, 50 mL of 0.08 mM CAS, 10 mL of 1 mM filtered FeCl_3_ × 6H_2_O in 10 mM HCl and 40 mL of 2 mM CTAB (CAS, FeCl_3_ and CTAB were mixed before adding to solution). Plates were incubated at 30 °C for 1 h and the intensity and diameter of the orange halo were compared. The experiment was performed two times for five replicates for each strain.

### 4.12. RNA Extraction

Bacterial RNA was extracted using the Total RNA Mini Plus RNA extraction kit (A&A Biotechnology, Gdynia, Poland) according to the manufacturer’s instructions. The quantity and quality of the RNA samples were confirmed by measurement of absorbance at 260 nm and evaluation of A260/A280 (~2) and A260/A230 (>2) ratios, and by agarose gel electrophoresis. Samples of 5 µg of RNA were subjected to DNase treatment (A&A Biotechnology, Poland) by incubation of 20 µL reaction mixtures in the presence of DNase (1U/µL) at 37 °C for 25 min followed by incubation at 75 °C for 10 min. The samples served as a template for the reverse transcription reaction.

### 4.13. Reverse Transcription

cDNA was transcribed from 1.5 µg of RNA with the use of RevertAid First Strand cDNA Synthesis Kit (Thermo Fisher Scientific, Waltham, Massachusetts, USA), according to manufacturer’s protocol. Obligatory step of denaturation of RNA with random hexamer primers mixture at 65 °C for 5 min was added.

### 4.14. Quantitative Real-Time PCR (qPCR)

qPCR analysis was performed as described in [23]. Briefly, diluted cDNA samples in a 1:2 ratio were used as qPCR templates. The qPCR reactions were carried out using the LightCycler 96 instrument (Roche Diagnostics, Rotkreuz, Switzerland). Primer3 software was used to design primers [71] (Table 4). Ten-fold dilution series of genomic DNA templates isolated from *D. solani* IPO 2222 were used to estimate the amplification efficiency of each pair of primers. qPCR reaction was carried out with FastStart Essential DNA Green Master (Roche Diagnostics, Rotkreuz, Switzerland). A 20 µL qPCR reaction mixture contained 0.5 µL of cDNA, 3–4.5 pmol of forward and reverse primers, 10 µL of PCR Mix. Thermal cycling parameters were as follows: preincubation at 95 °C for 5 min; 35–50 cycles of amplification and quantitation at 95 °C for 15 s, 62 °C for 20 s and 72 °C for 16 s. At the end of each cycle, melting curve analysis was performed (95 °C for 10 s, 65 °C for 60 s and 97 °C for 1 s). All qPCR reactions were performed for three biological replicates, with three technical repeats, negative no template control (NTC) and no-reverse transcriptase (NRT) controls. Cq (quantification cycle) values were averaged. The 16s rRNA gene was selected for normalization as it showed stability under all tested conditions. Pfaffl-ΔΔCT method with correction for PCR efficiency was used for the determination of the relative expression of the *lon* gene [72]. Statistical analysis was performed with the use of REST2009 software (v. 2009, Qiagen, Hilden, Germany) [73,74].

### 4.15. Protein Electrophoresis and Immunodetection

SDS page electrophoresis and Western blotting were performed as described in [75,76]. Then, 7.5% polyacrylamide gels were used. Briefly, the Lon protein was detected with the anti-*Escherichia coli* Lon rabbit antibodies (#40219-T24, Sino Biological Inc., Beijing, China) at dilution 1:2000 followed by incubation with HRP conjugated secondary anti-rabbit antibodies (#31462 Thermo Fisher) diluted 1:50,000. Chemiluminescent signal was developed using a luminol/ p-coumaric acid (Carl Roth GmbH + Co. KG) mix (4 mL of 1.41 mM luminol, 400 µL of 6.7 mM p-coumaric acid in DMSO, 4 µL of 30% H_2_O_2_) and was recorded by Azure Biosystems c600 (Dublin, California, USA) imaging system.

### 4.16. Sample Preparation for Mass Spectrometry

The stationary growth phase cultures of *D. solani* cultivated in M63Y were subjected to high-temperature stress. Briefly, the cultures were transferred from 30 °C to 40 °C and incubated for 30 min with shaking. For control conditions, bacteria were cultivated at 30 °C. Five biological replicates of each strain were pooled and centrifuged (7000× *g*, 2 min). The pellets were lysed with the solution containing 4% SDS, 100 mM Tris/HCl pH 7.6, 0.1 M DTT (lysis solution) and incubated at 95 °C for 10 min. After cooling, cold acetone was added to the solution to precipitate the released proteins. The samples were incubated at -20 °C for about 2 h and then centrifuged for 20 min 20,000× *g*. The supernatant was decanted and the precipitate dried. The pellet was then dissolved in 8 M urea in 0.1 M Tris/HCl pH 8.5 [77].

### 4.17. Protein Digestion

First, the protein concentration was measured by measuring absorbance at 280 nm (MultiskanTM Thermo, Waltham, Massachusetts, USA) using the µDrop plate. Digestion was carried out according to the standard Filter Aided Sample Preparation (FASP) procedure [77]. Then, 100 µg of protein was used for each digestion and the procedure was carried out using microcons with 10 kDa mass cut-off membrane. Generated tryptic peptides were desalted with StageTips according to the protocol described by Rappsilber [78]. For each desalting step, 10 µg of the peptide was taken and desalted on StageTip containing three layers of 3 M Empore C18 exchange disks.

### 4.18. Liquid Chromatography and Mass Spectrometry

LC-MS/MS analysis was performed with the use of a Triple TOF 5600+ mass spectrometer (SCIEX Framingham, MA) coupled with the Ekspert MicroLC 200 Plus System (Eksigent, Redwood City, California, USA). All chromatographic separations were performed on the ChromXP C18CL column (3 µm, 120 Å, 150 × 0.3 mm). The chromatographic gradient for each IDA and SWATH runs was 3.5–20% B (solvent A 0% aqueous solution 0.1% formic acid, solvent B 100% acetonitrile 0.1% formic acid) in 60 min. The whole system was controlled by the SCIEX Analyst TF 1.7.1 software (version 1.7.1, Framingham, MA, USA).

### 4.19. SWATH Mass Spectrometry Experiments

All samples were acquired in triplicates. Experiments were performed in a looped product ion mode.

A set of 25 transmission windows (variable wide) was constructed and covered the precursor mass range of 400–1200 *m*/*z*. The collision energy for each window was calculated for +2 to +5 charged ions centered upon the window with a spread of two. The SWATH-MS1 survey scan was acquired in high sensitivity mode in the range of 400–1200 Da in the beginning of each cycle with the accumulation time of 50 ms, and SWATH-MS/MS spectra were collected from 100 to 1800 *m*/*z* followed by 40 ms accumulation time high sensitivity product ion scans, which resulted in the total cycle time of 1.11 s.

### 4.20. Data Analysis

Database search was performed with ProteinPilot 4.5 software (Sciex, v.4.5 AB, Framingham, MA, USA) using the Paragon algorithm against the UNIPROT *Dickeya solani* database with an automated false discovery rate, and standard parameters [79,80]. Next, a spectral library was created with the group file data processing in PeakView v. 2.2 (Sciex), with parameters as described in detail by Lewandowska [79]. Files from SWATH experiments for each sample were downloaded to PeakView (Sciex, v.2.2, Framingham, MA, USA) software and processed with the previously established library. Resulting data were exported to the .xml file and exported to Marker View software. All data were normalized using total area sums (TAS) approach, grouped as wild type and tested samples and *t*-test was performed. Samples were compared to each other, coefficient of variation (CV%) was calculated, and proteins with a *p*-value lower than 0.05 with fold change 2 were considered as differentially expressed in examined samples. The mass spectrometry proteomics data have been deposited to the ProteomeXchange Consortium via the PRIDE [81] partner repository with the dataset identifier PXD018297.

## Figures and Tables

**Table 1:** Summary of proteins whose level depends on Lon protease.

Protein		30 °C		40 °C	
Accession Number	Name	x-Fold	Log_2_x-Fold	x-Fold	Log_2_x-Fold
**Motility**					
• Downregulated					
A0A2K8VVE7_9GAMM	Flagellin	0.35	−0.46		
A0A2K8VVK5_9GAMM	Protein phosphatase CheZ	0.41	−0.39		
A0A2K8W5V2_9GAMM	Methyl-accepting chemotaxis protein I (Serine chemoreceptor protein)	0.24	−0.63	0.21	−0.67
A0A2K8VVH9_9GAMM	Signal transduction histidine kinase CheA			0.34	−0.46
A0A2K8VXS1_9GAMM	Methyl-accepting chemotaxis protein I (Serine chemoreceptor protein)			0.43	−0.37
A0A2K8W5V2_9GAMM	Methyl-accepting chemotaxis protein I (Serine chemoreceptor protein)	0.24	−0.63		
A0A2K8VVG7_9GAMM	Flagellar motor switch protein FliG			0.46	−0.34
A0A2K8VVJ6_9GAMM	Positive regulator of CheA protein activity (CheW)			0.35	−0.45
**Iron metabolism**					
• Downregulated					
A0A2K8VW36_9GAMM	Ferrichrome-iron receptor	0.50	−0.30	0.27	−0.56
A0A2K8VW52_9GAMM	2,3-dihydroxybenzoate-AMP ligase enterobactinsiderophore	0.30	-0.52	0.15	-0.82
A0A2K8W494_9GAMM	Nonspecific DNA-binding protein Dps/Iron-binding ferritin-like antioxidant protein/Ferroxidase			0.47	−0.32
A0A2K8VUB7_9GAMM	Ferrous iron transport protein B			0.45	−0.34
A0A2K8VW26_9GAMM	Isochorismatase enterobactin siderophore/Apo-aryl carrier domain of EntB			0.4	−0.34
A0A2K8VW34_9GAMM	Isochorismate synthase enterobactin siderophore	0.48	−0.32		
A0A2K8VW22_9GAMM	Enterobactin synthetase component F, serine activating enzyme			0.36	−0.44
• Upregulated					
A0A2K8W4W8_9GAMM	Achromobactin biosynthesis protein AcsASiderophoresynthetase superfamily, group B	7.58	0.88	3.044	0.48
A0A2K8VWX6_9GAMM	Iron-sulfur cluster insertion protein ErpA			4.90	0.69
A0A2K8W3W2_9GAMM	Ferric uptake regulation protein			9.87	0.99
A0A2K8VWQ7_9GAMM	Iron-sulfur cluster assembly scaffold protein IscU			3.67	0.56
A0A2K8W4W2_9GAMM	Achromobactin biosynthesis protein AcsD Siderophore synthetase superfamily, group A	2.77	0.44		
**Stress-Related**					
• Downregulated					
A0A2K8VUY7_9GAMM	Phage shock protein A	0.31	−0.51	0.35	−0.45
A0A2K8VZS1_9GAMM	Small heat shock protein IbpA	0.45	−0.35		
A0A2K8VZU2_9GAMM	Universal stress protein			0.49	−0.31
A0A2K8VUY1_9GAMM	Phage shock protein B OS=Dickeya solani			0.38	−0.41
• Upregulated					
A0A2K8VTF5_9GAMM	Protease II	4.44	0.65	2.94	0.47
A0A2K8VX21_9GAMM	Protein RecA			2.65	0.42
A0A2K8W3I9_9GAMM	ATP-dependent Clp protease proteolytic subunit			2.00	0.30
A0A2K8W3U6_9GAMM	Cold shock protein CspE			2.91	0.46
A0A2K8VZ71_9GAMM	Cold shock protein CspG			17.27	1.24
A0A2K8W1Q0	Osmotically inducible protein OsmY			2.50	0.40
A0A2K8VUA2_9GAMM	Protease HtpX			2.22	0.35
• Differentially expressed					
A0A2K8W260_9GAMM	Periplasmic protein related to spheroplast formation	0.40	−0.40	2.79	0.45
**Transport**					
• Downregulated					
A0A2K8W052_9GAMM	Phosphate-binding protein PstS	0.48	−0.32		
A0A2K8VVW1_9GAMM	Histidine ABC transporter, histidine-binding periplasmic protein HisJ	0.47	−0.33		
A0A2K8VU80_9GAMM	N-acetylneuraminic acid outer membrane channel protein NanC	0.27	−0.56		
A0A2K8VTK7_9GAMM	Oligopeptide ABC transporter, periplasmic oligopeptide-binding protein OppA			0.46	−0.34
A0A2K8VWQ4_9GAMM	Periplasmic substrate-binding transport protein			0.49	−0.31
A0A2K8W2K3_9GAMM	Inositol transport system sugar-binding protein	0.41	−0.39		
A0A2K8W3K7_9GAMM	Efflux pump membrane transporter	0.37	−0.43		
A0A2K8VZR0_9GAMM	Dipeptide-binding ABC transporter, periplasmic substrate-binding component			0.39	−0.41
A0A2K8W417_9GAMM	Cobalt/zinc/cadmium efflux RND transporter, membrane fusion protein, CzcB family			0.47	−0.337
A0A2K8VSS7_9GAMM	Methionine ABC transporter substrate-binding protein			0.44	−0.36
• Upregulated					
A0A2K8W0I4_9GAMM	Xylose ABC transporter, periplasmic xylose-binding protein XylF	3.06	0.49	2.85	0.45
A0A2K8VXC1_9GAMM	L-proline glycine betaine binding ABC transporter protein ProX	2.25	0.35	2.46	0.39
A0A2K8W3M0_9GAMM	Lead, cadmium, zinc and mercury transporting ATPase			3.14	0.50
**Metabolism**					
• Downregulated					
A0A2K8W021_9GAMM	ATP synthase subunit delta	0.47	−0.33		
A0A2K8W4F2_9GAMM	Glutamate-1-semialdehydeaminotransferase	0.10	−1.01	0.16	−0.806
A0A2K8W580_9GAMM	Enoyl-acyl-carrier-protein reductase NADPH	0.38	−0.42		
A0A2K8W1R7_9GAMM	Alkyl hydroperoxide reductase protein C	0.40	−0.40		
A0A2K8VTW6_9GAMM	NAD(P) transhydrogenase subunit alpha	0.47	−0.33		
A0A2K8W040_9GAMM	ATP synthase epsilon chain	0.23	−0.64		
A0A2K8W444_9GAMM	6-phosphogluconolactonase	0.40	−0.40		
A0A2K8VVU5_9GAMM	NADH-quinone oxidoreductase			0.46	−0.33
A0A2K8VV27_9GAMM	Endo-1,4-beta-xylanase A	0.230	−0.53	0.13	−0.89
A0A2K8VXQ3_9GAMM	PTS system, cellobiose-specific IIB component			0.45	−0.35
A0A2K8W121_9GAMM	Biotin carboxyl carrier protein of acetyl-CoA carboxylase	0.41	−0.39		
A0A2K8W3J3_9GAMM	Cytochrome O ubiquinol oxidase subunit I			0.47	−0.32
A0A2K8VTY0_9GAMM	Superoxide dismutase [Cu-Zn]			0.38	−0.42
A0A2K8VU81_9GAMM	Sugar-binding protein	0.47	−0.33	0.37	−0.43
A0A2K8W4J6_9GAMM	Putative l-lactate dehydrogenase, Iron-sulfur cluster-binding subunit YkgF			0.36	−0.44
A0A2K8VWF7_9GAMM	Peptidyl-prolyl *cis*-*trans* isomerase			2.31	0.36
A0A2K8VT63_9GAMM	ATP phosphoribosyltransferase			0.45	−0.35
A0A2K8VVY8_9GAMM	Acetyl-coenzyme A carboxylase carboxyl transferase subunit beta			0.42	−0.38
A0A2K8VX73_9GAMM	Phosphoheptose isomerase			0.28	−0.55
A0A2K8W077_9GAMM	Bifunctional polymyxin resistance protein ArnA			0.20	−0.71
• Upregulated					
A0A2K8VTR1_9GAMM	3-hydroxypropionate dehydrogenase	4.17	0.62	4.24	0.63
A0A2K8VV57_9GAMM	Thioredoxin/glutathione peroxidase BtuE	3.29	0.52	5.75	0.76
A0A2K8VXQ4_9GAMM	Putative phosphatase/kinase	6.94	0.84	10.73	1.03
A0A2K8VTR5_9GAMM	SAM-dependent methyltransferase YafE (UbiE-like protein)	4.13	0.62	4.38	0.64
A0A2K8W193_9GAMM	Glyoxalase	2.42	0.38		
A0A2K8VYC9_9GAMM	Fructose-bisphosphate aldolase class II			2.80	0.45
A0A2K8W3S4_9GAMM	Thiol peroxidase, Bcp-type			2.57	0.41
A0A2K8VV22_9GAMM	Glutaredoxin			2.77	0.44
A0A2K8W3N7_9GAMM	Stomatin/prohibitin-family membrane protease subunit YbbK	2.66	0.43		
A0A2K8VXG5_9GAMM	Adenylate cyclase	2.37	0.38		
A0A2K8W3S1_9GAMM	Glycoprotein/polysaccharide metabolism	14.75	1.17	3.187	0.507
A0A2K8VVX6_9GAMM	Phosphatase YfbT			2.04	0.31
A0A2K8VYD6_9GAMM	Biosynthetic arginine decarboxylase			3.62	0.56
A0A2K8VSW1_9GAMM	Soluble aldose sugar dehydrogenase, PQQ-dependent PE = 4 SV = 1			2.38	0.38
A0A2K8VXS7_9GAMM	Aminotransferase	2.36	0.37		
A0A2K8VXE7_9GAMM	Sulfite reductase [NADPH] flavoprotein alpha-component	2.03	0.31		
A0A2K8VY35_9GAMM	3-isopropylmalate dehydratase large subunit			2.21	0.34
A0A2K8W254_9GAMM	Phosphopentomutase			2.60	0.41
A0A2K8VT52_9GAMM	Phosphoserine aminotransferase	2.47	0.39		
A0A2K8W0R3_9GAMM	ADP-l-glycero-d-manno-heptose-6-epimerase	4.45	0.65	5.20	0.72
A0A2K8VY36_9GAMM	3-isopropylmalate dehydratase small subunit			2.51	0.40
A0A2K8VWH1_9GAMM	Methylglyoxal synthase	2.27	0.36	2.85	0.45
• Differentially expressed					
A0A2K8W2T8_9GAMM	Phosphotransferase system, phosphocarrier protein HPr	0.49	−0.31	2.66	0.42
A0A2K8VYF1_9GAMM	Epimerase domain-containing protein	2.34	0.37	0.18	−0.75
A0A2K8W293_9GAMM	Thiol:disulfide interchange protein	0.46	−0.34	2.13	0.33
**Virulence**					
• Upregulated					
A0A2K8VU37_9GAMM	Various polyols ABC transporter, permease component 2			2.75	0.44
A0A2K8VUF2_9GAMM	Harpin hrpN (Harpin-Ech)			2.28	0.36
A0A2K8VUE5_9GAMM	Hrp pili protein hrpA (TTSS pilin hrpA)			10.64	1.03
**Transcription/Translation**					
• Downregulated					
A0A2K8VT33_9GAMM	Serine-tRNA ligase			0.47	−0.33
A0A2K8VW75_9GAMM	JmjC domain-containing protein			0.48	−0.32
• Upregulated					
A0A2K8VZG3_9GAMM	Transcriptional (Co)regulator CytR	3.47	0.54		
A0A2K8VZY6_9GAMM	DNA-directed RNA polymerase subunit omega			2.45	0.39
A0A2K8W0W8_9GAMM	50S ribosomal protein L7/L12			2.11	0.32
A0A2K8VUA3_9GAMM	Translation initiation factor 3			2.69	0.43
A0A2K8VX20_9GAMM	Ribosome hibernation protein YfiA			13.75	1.14
A0A2K8W224_9GAMM	50S ribosomal protein L27			2.03	0.31
A0A2K8W3J6_9GAMM	50S ribosomal protein L31 type B			3.42	0.53
A0A2K8VWY3_9GAMM	RNA polymerase-binding transcription factor DksA			2.07	0.32
A0A2K8VY97_9GAMM	ABC transporter, ATP-binding protein	2.45	0.39	2.04	0.31
A0A2K8VYE8_9GAMM	RNA-binding protein Hfq			2.70	0.43
A0A2K8VYF1_9GAMM	Epimerase domain-containing protein	2.34	0.37	0.18	−0.75
**Others**					
• Downregulated					
A0A2K8W376_9GAMM	UPF0325 protein D083_3591	0.36	−0.45		
A0A2K8W2W2_9GAMM	IncI1 plasmid conjugative transfer protein TraF	0.30	−0.53	0.26	−0.58
A0A2K8W4R1_9GAMM	Uncharacterized protein	0.45	−0.34	0.33	−0.48
A0A2K8VTN2_9GAMM	Uncharacterized protein	0.41	−0.38		
A0A2K8VV11_9GAMM	Major outer membrane lipoprotein			0.50	−0.30
• Upregulated					
A0A2K8W3L1_9GAMM	Putative membrane protein OS=Dickeya solani	175.34	2.24	117.58	2.07
A0A2K8VWZ4_9GAMM	S-ribosylhomocysteinelyase			2.03	0.31
A0A2K8VZT1_9GAMM	Putative membrane protein			7.12	0.85
A0A2K8VTR0_9GAMM	Putative secreted protein			5.63	0.75
A0A2K8VT84_9GAMM	Uncharacterized protein	5.23	0.72	6.61	0.82
• Differentially expressed					
A0A2K8W4I6_9GAMM	Uncharacterized protein	4.19	0.62	0.33	−0.48

**Table 2:** Bacterial strains and plasmids used in this study.

**Strain**	**Genotype**	**Reference or Source**
*Escherichia coli* DH5α	F– φ80lacZΔM15 Δ(lacZYA-argF)U169 recA1 endA1 hsdR17(rK–, mK+) phoA supE44 λ– thi-1 gyrA96 relA1	[58]
*Escherichia coli* DH5α *pir*	sup E44 ΔlacU169 (ΦlacZΔM15) recA1 endA1 hsdR17 thi-1 gyrA96, relA1 λpir phage lysogen	[59]
*Escherichia coli* MFD *pir*	MG1655 RP4-2-Tc::[ΔMu1::aac(3)IV-ΔaphA-Δnic35-ΔMu2::zeo] ΔdapA::(erm-pir) ΔrecA	[60]
*Dickeya solani* IPO 2222	WT	[61]
*D. solani* IPO 2222 Δ*lon*	Δ*lon*	This work
*D. solani* IPO 2222 Δ*lon/lon*	Δ*lon/lon*	This work
**Plasmids**	**Feature**	**Reference or Source**
pDOC-C	pEX100T, Sce1 -Sce1 *sacB* Amp^R^	[62]
pDOC-K	pEX100T, Sce1-Kan^R^ -Sce1 *sacB* Amp^R^	[62]
pACBSCE	I-Sce1 λ-Red Cm^R^	[62]
pDFDOC-C-lon	pDOC-C Sce1-Kan^R^-Sce1	This work
pRE112	pRE107 cm^R^ *sacB*	[63]
pmScarlet	pMB1 ori mScarlet Amp^R^	[64]
pLonScar	pRE112 *lon mScarlet*	This work

Please define all abbreviations in table footer, if appropriate.

**Table 3:** List of primers and their characteristics.

Primer	Primer Sequence 5′-3′	Amplified DNA
lonkan L	CAGGGTACCTTCCCTTAACCTGGCGGAAACGAAACTAAGAGAGAGCTCTGACCGGTCAATTGGCTGGAG	kanamycin resistance gene with added sequences flanking the *D. solani lon* gene amplified from pDOC-K
lonkan R	GCACACTCGAGCCAGCCTTTTT TTCTCAGTGGTTTTTGCGATAGGTCACTAATATCCTCCTTAGTTCC	
lonsolani L	CGATTACCTATAGGCGAAACC	lon and kanamycin resistance gene amplified from *D. solani* and *D. solani Δ lon* gDNA, respectively
lonsolani R	CAGGCTCAACAGTGCTCTAAC	
1 L	AGTGAACTGCATGAATTCCCGTTGATCCAGATCTTGCGCGA	500 bp upstream from the start codon of *lon* gene amplified from *D. solani* gDNA
1 R	GTTCGGAACGCTCAGGGTTCATAGAGCTCTCTCTTAGTTTCGTTTCC	
2 lon L	ATGAACCCTGAGCGTTCCGAA	*lon* gene amplified from *D. solani* gDNA
2 lon R	CACGTTTCACTTTCCGGGTTCCTATTTTTTGGCTACCGACTTCAC	
3 scarlet L	GAGACCCGGAAAGTGAAAACGTG	*mScarlet* gene amplified from pmScarlet
3 scarlet R	TTACCGCCTTTGAGTGAGCTG	
4 L	CAGCTCACTCAAAGGCGGTAATGACCTATCGCAAAAACCAC	500 bp downstream from the stop codon of *lon* gene amplified from *D. solani* gDNA
4 R	ATGCGATATCGAGCTCTCCCAAAACCGTCCCACCTCAGATT	

**Table 4:** Characteristics of primers used in gene expression analysis.

	FWD Primer Sequence (5′-3′)	REV Primer Sequence (5′-3′)	Amplicon Length [bp]	PCR Efficiency	R^2^	Concentration [µM]
*lon*	TGGTCATTCCGTTGTTTGTTGGTC	CATCCGTTGAGGCTTCTTTCTGTG	111	1.97	1.0	0.3
16S rRNA	GCTCGTGTTGTGAAATGTTGGGTT	GCAGTCTCCCTTGAGTTCCCAC	94	1.96	1.0	0.225

## Supplementary Materials

The following are available online at https://www.mdpi.com/1422-0067/21/10/3687/s1, Figure S1: Verification of the constructed Δ*lon/lon* strain, Table S1: SWATH-MS raw data, Figure S2: Ability of *D. solani* Δ*lon* to cause maceration of plant tissues: (A) potato tubers (B) chicory leaves and (C) Chinese cabbage leaves.

## Abbreviations

CAS	casamino acids
CFU	colony forming units
CMC	carboxymethyl cellulose
C_q_	quantification cycle
DAP	diaminopimelic acid
LA	Luria Agar
MFD	Mu-free donor
OD	optical density
PGA	polygalacturonic acid
PCWDE	plant cell wall degrading enzymes
PQCS	protein quality control system
SRP	soft rot Pectobacteriaceae
SWATH-MS	Sequential Window Acquisition of All Theoretical Mass Spectra
T1SS	type I secretion system
T2SS	type II secretion system
T3SS	type III secretion system
WT	wild-type
